# Retrospective Evaluation of Survival and Prognostic Factors in Immune Thrombocytopenia: A Single-Center and Cross-Sectional Study

**DOI:** 10.3390/medicina60071153

**Published:** 2024-07-17

**Authors:** Gökhan Pektaş, İbrahim Asaf Uncu, Yelda Dere, Şeyma Öncü, Merve Becit Kızılkaya, Gökhan Sadi, Mehmet Bilgehan Pektaş

**Affiliations:** 1 Division of Hematology, Faculty of Medicine, Muğla Sıtkı Koçman University, 48000 Muğla, Türkiye; gokhanpektas@gmail.com (G.P.); uncuasaf@gmail.com (İ.A.U.); 2 Department of Medical Pathology, Faculty of Medicine, Muğla Sıtkı Koçman University, 48000 Muğla, Türkiye; yeldadere@mu.edu.tr; 3 Department of Medical Pharmacology, Faculty of Medicine, Afyonkarahisar Health Sciences University, 03200 Afyonkarahisar, Türkiye; seyma.oncu@afsu.edu.tr; 4 Department of Toxicology, Faculty of Pharmacy, Afyonkarahisar Health Sciences University, 03200 Afyonkarahisar, Türkiye; merve.becit@afsu.edu.tr; 5 Department of Biology, K.O. Science Faculty, Karamanoglu Mehmetbey University, 70100 Karaman, Türkiye; sadigokhan@gmail.com

**Keywords:** immune thrombocytopenia, IVIg, eltrombopag, rituximab

## Abstract

*Background and Objectives:* Immune thrombocytopenia (ITP) is an autoimmune disease characterized by the autoantibody-mediated destruction of platelets. The treatment of ITP aims to maintain a sufficient platelet count to prevent bleeding. First-line treatment options include corticosteroids and intravenous immunoglobulin (IVIg), while second-line treatments include splenectomy, rituximab and other immunosuppressive agents, and thrombopoietin (TPO) receptor agonists. This study aims to discuss the treatment methods and results from 100 patients with ITP at the Muğla Training and Research Hospital through a pharmacological approach. *Materials and Methods:* Demographic characteristics, clinical findings, bone marrow aspiration and biopsy results, and treatments and treatment responses at the time of diagnosis of the 100 patients with ITP who were treated and followed up in the period 2015–2023 were evaluated retrospectively. *Results:* In the third month after treatment, the overall response percentage was 100% in patients who received steroids only and 88% in patients who received IVIg treatment alone or in combination with steroids (*p* > 0.05). The most preferred second-line treatments were splenectomy (41%), eltrombopag (26%), and rituximab (10%). Bone marrow biopsy was performed in 54% of patients, where 35.1% showed increased megakaryocytes, 44.4% adequate megakaryocytes, and 14.8% decreased megakaryocytes. It is noted that eltrombopag and rituximab, in particular, yield higher complete remission rates than immunosuppressive drugs. *Conclusions:* Considering the side effects of immunosuppressive medications, IVIg, splenectomy, and steroid therapy, the use of new agents such as eltrombopag, which are easily tolerated and have a lower risk of side effects, is expected to increase.

## 1. Introduction

Primary immune thrombocytopenia (ITP) is an autoimmune bleeding disorder characterized by isolated thrombocytopenia in the absence of other etiologies [1]. In primary ITP, increased platelet destruction and platelet production are observed. Secondary ITP denotes all forms of immune-mediated thrombocytopenia other than primary (e.g., due to drug use, malignancy, and viral infections) [2]. Primary ITP accounts for approximately 80% of cases, while secondary ITP accounts for 20% [3]. Although ITP is a rare disease, the prevalence of the disease in adults has been reported to be 1.6–3.9/100,000 per year [4,5]. Through the examination of large-scale studies, it was found that the incidence of ITP was increased in young women and elderly men [6]. On the contrary, some studies did not find any significant relationship with predisposing factors such as age, gender, or family history [7]. In the pathogenesis of primary ITP, thrombopoiesis is suppressed, and the antibody-mediated and cellular destruction of platelets occurs [8]. The dysfunction of megakaryocytes and low thrombopoietin levels play a role in decreasing platelet production [9]. The most important criterion in the diagnosis of ITP is a low platelet count (<100 × 10^9^/L) that cannot be explained by another reason [10]. ITP patients typically have no other physical or laboratory findings to confirm the diagnosis. Megakaryocytes are responsible for platelet production and so the number and morphology of megakaryocytes in the bone marrow reflect the destruction and production of platelets [11]. Even though there is no definitive diagnostic test for ITP, morphological examination of megakaryocytes has come to the fore in the recommendations provided by guidelines [12]. Before being diagnosed with ITP, many patients are asymptomatic or may present to the emergency room with only minor mucocutaneous bleeding. However, it has been reported that severe bleeding may occur in 5–6% of patients, resulting in decreased quality of life, morbidity, or mortality [13]. Bleeding risks, especially hemorrhage and intracranial hemorrhage, are the most serious complications for patients diagnosed with ITP. Compared to the general population, there is a 1.5-fold increase in the incidence of cardiovascular events and a 1.7-fold increase in venous thromboembolism in ITP patients, and this risk increases further with the use of antiplatelet or anticoagulant drugs [14]. In a 10-year multicenter study conducted on newly diagnosed ITP patients, the rate of bleeding events was 53%, the all-cause mortality rate was 11%, and the ITP-related mortality rate was found to be 3% [15]. While treatment of primary ITP focuses on reducing platelet destruction and stimulating platelet production, it also focuses on resolving the underlying cause or disorder in secondary ITP. Treatments such as corticosteroids, intravenous immune globulin (IVIg), and/or platelet transfusions are administered alone or in combination [16]. There have been advances in the treatment of ITP in recent years, including the use of rituximab, immunosuppressants, and thrombopoietin receptor agonists (TPO-Ras) such as eltrombopag. These treatments have been emphasized in the American Society of Hematology’s newest guide for the treatment of ITP. Another option, splenectomy, is recommended when there is no response to treatments after a certain period [12]. Treatment should be adjusted according to the presence of bleeding, increase in the desired platelet count, side effects of treatment, and patient preferences [17]. The aim of ITP treatment is to prevent serious bleeding complications, maintain a platelet level of at least 20–30 × 10^9^/L, and minimize the toxicity of the drugs [18]. As approximately one-third of patients do not respond to steroid treatment, they must receive multiple monotherapy or combination treatments until a permanent cure is achieved. This leads to an increased risk of side effects such as bleeding or thrombosis and a loss of time [19]. Studies have examined the response to steroids in ITP treatment in terms of platelet number and morphology [8,20,21]. However, there is a need for studies examining the combined effects of agents used in first- and second-line treatment. Based on this information, the purpose of the study is to examine the correlation between first-line (steroids and IVIg) and second-line (rituximab, TPO-Ras, and immunosuppressive agents) treatments in patients diagnosed with ITP, in terms of (a) platelet count, (b) megakaryocyte morphology, and (c) cure achieved in the 3rd and 12th months of treatment.

## 2. Materials and Methods

### 2.1. Patient Recruitment

A single-center, cross-sectional study was performed after approval was obtained from the local Ethics Committee of the Muğla Sıtkı Koçman University (2022/92). The study was conducted on 100 patients diagnosed with ITP in the Muğla Training and Research Hospital from 2015 to 2023.

### 2.2. Study Population

Primary ITP was defined as a platelet count ≤100 × 10^9^/L, with no evidence of other causes of thrombocytopenia. Secondary thrombocytopenia, such as that due to sepsis, drug-induced thrombocytopenia, hematological disorders, and portal hypertension, was excluded. The inclusion criteria included all patients with primary ITP over 18 years, while the exclusion criteria were under 18 years of age, pregnant during ITP monitoring, and breastfeeding volunteers.

### 2.3. Data Collection

Demographic characteristics of the patients (age, gender), ITP diagnosis date, ITP type (primary–secondary), survival status, Charlson Comorbidity Index (CCI) score, bleeding status, platelet transfusion, and drug use at diagnosis were recorded from the patients’ file information. According to their CCI scores, the severity of comorbidity was categorized into three grades: mild with a CCI score of 1–2, medium with a CCI score of 3–4, and severe with a CCI score greater than or equal to 5 [22]. Corticosteroid and IVIg are the first-line treatment options; in the second and third stages, splenectomy, rituximab, TPO-receptor agonist eltrombopag, danasin, cyclosporine, cyclophosphamide, azathioprine, and mycophenolate fomethyl information were obtained from the file information. Treatment responses were evaluated at the end of the 3rd and 12th months.

ITP stages have been redefined: (a) newly diagnosed ITP covers the first 3 months from diagnosis. (b) Persistent ITP covers 3–12 months from diagnosis. This includes cases that are months away and do not go into spontaneous remission or cannot remain in remission when treatment is stopped. (c) Chronic ITP covers ITP lasting 12 months or more. 

Severity of ITP: cases with clinically significant bleeding findings are classified as severe ITP. Definition of the response to treatment: the absence of bleeding findings is required for response. (a) Complete response: platelet count > 100 × 10^9^/L. (b) Response: cases with a platelet count of 30–100 × 10^9^/L and at least twice the initial platelet count. (c) Unresponsive: platelet count <30 × 10^9^/L and not reaching twice the initial platelet count.

### 2.4. Pathological Evaluation in Bone Marrow

For differential diagnosis, histological slides of bone marrow biopsy samples and aspirates from 54 patients with severe primary ITP were sent to pathology. The pathologist was asked to describe the ITP bone marrow and evaluate the megakaryocyte number, morphology, and distribution. No other pathology was found in the biopsy samples through immunohistochemical staining. Histologically, the pathologist reported increased megakaryocyte numbers and stumped megakaryocytes as being compatible with ITP.

### 2.5. Statistical Analysis

Descriptive data are presented as number (*n*), percentage (%), and mean (standard deviation) values. The conformity of the data to the normal distribution was checked using the Shapiro–Wilk test. Non-parametric variables are presented as the median and interquartile range. The relationships between dependent and independent variables were evaluated with the chi-square test, *Fisher’s Exact* test, *Mann–Whitney U*-test, and *Kruskal–Wallis* test. Data were analyzed using SPSS v. 24.0.0.1 (SPSS Inc., Chicago, IL, USA) statistical software. A *p*-value less than 0.05 was considered statistically significant.

## 3. Results

The mean age of the 100 patients included in the study was 52.9 ± 19.5 years (minimum: 20; maximum: 93), and 43% of the patients were female. The CCI score was found to be 5 or above in 23% of the patients. The number of patients diagnosed with ITP in 4 years or less was 47.97% of the patients who had a primary diagnosis of ITP. At the time of diagnosis, 95% of the patients had mild bleeding. Blood transfusion was performed in four patients, and splenectomy was performed in 41 patients (Table 1). When drug use at the time of diagnosis was examined, 41 patients were using antiplatelet–anticoagulant drugs. As the first-line treatment, IVIg was used in 86% of the patients and methylprednisolone was used in 99%. The number of patients receiving second-line treatment was 32. Eltrombopag was used in 26 of these patients, rituximab was used in 10, and danasin was used in 4 (Table 1). Cyclosporine, cyclophosphamide, azathioprine, and mycophenolate mofetil were among the other drugs used. A combination of one drug was used in 22 patients receiving second-line treatment, two drugs were used in 8 patients, and three drugs were used in combination in 2 patients. At the end of the 3rd month, 58% of the patients had complete remission, and 23% had partial remission. At the end of the 12th month, 70% had complete and 17% had partial remission (Table 1). Mortality occurred in 4% of patients. The age range of these four patients was 76–88 years, and their CCI was calculated as 7 and above (severe). The median survival of these patients after diagnosis of ITP was one year. There was partial bleeding at the time of the ITP diagnosis. While remission was complete in all of them in the 3rd month, partial remission was achieved in only one patient, and complete remission was achieved in three patients at the 12th month. The median platelet level of the patients was determined to be 6 × 10^3^/mL (Q1–Q3: 3–17.75 × 10^3^/mL) at the time of first admission, when ITP was diagnosed. Platelet levels increased to 148.5 × 10^3^/mL (Q1–Q3: 54.0–264.25 × 10^3^/mL) in the third month and 175 × 10^3^/mL (Q1–Q3: 69.25–286 × 10^3^/mL) in the 12th month. The hemogram laboratory findings of the patients in the 3rd and 12th months are presented in Table 2. It was found that 18.6% (*n* = 16) of 86 patients receiving IVIg treatment did not have complete or partial remission at the 3rd month and 11.6% at the 12th month (Table 3). In the 3rd month, remission was complete in 59.3% (*n* = 51) of those receiving IVIg treatment and partial in 18.6% (*n* = 16). In the 12th month, these rates were found to be 69.7% (*n* = 60) and 15.1% (*n* = 13), respectively. No significant difference was detected in terms of remission between patients who received and did not receive IVIg treatment (Table 3). When the effect of secondary drug use on remission was evaluated in the 3rd month, 11 (47.8%) of 23 patients using eltrombopag developed complete remission, 4 (17.4%) developed partial remission, and remission was unresponsive in 8 patients (34.8%). Of the 10 patients using rituximab, 6 had complete remission, and 2 had partial remission, while remission was unresponsive in 2 patients. While two of four patients using danasin had complete remission, one patient had partial remission, and one patient had no remission. Remission was complete in both patients using cyclosporine. There was no remission in one patient, each using cyclophosphamide and mycophenolate mofetil. Remission was complete in one patient using azathioprine (Table 4). When the effect of secondary drug use on remission was evaluated in the 12th month, 11 (47.8%) of 23 patients using eltrombopag developed complete remission, 6 (26.1%) developed partial remission, and remission was unresponsive in 6 patients (26.1%). Of the 10 patients using rituximab, 8 had complete remission, and 1 had partial remission, while remission was unresponsive in 1 patient. Of the four patients using danasin, one had partial remission, and three patients had no remission. Of the two patients using cyclosporine, one had complete remission, and the other had no remission. There was no remission in the single patients using azathioprine and mycophenolate mofetil, respectively. Remission was partial in one patient using cyclophosphamide (Table 4). The relationships between drug use and megakaryocyte morphology in first- and second-line treatments are presented in Table 5 and Table 6. Notably, 87.5% (*n* = 21) of patients with a normal megakaryocyte count, 92.3% (*n* = 12) of those with hypolobulation, and 88.9% (*n* = 8) of those with hyperlobulation were receiving IVIg treatment. Meanwhile, 45.8% (*n* = 11) of the patients with a normal megakaryocyte count, 61.5% (*n* = 8) of those with hypolobulation, and 55.6% (*n* = 5) of those with hyperlobulation were receiving second-line drug treatment. In both cases, nuclear fragmentation, micromegakaryocyte, hyperchromasia, paratrabecularity, and multilobulation were not observed.

## 4. Discussion

In this study, data from patients diagnosed with ITP in a training and research hospital (third-line therapy) were presented retrospectively. Drugs frequently used in primary and secondary care were examined, and their effects on laboratory and treatment responses were evaluated. The study was conducted in a small sample population.

While approximately 80% of the study group achieved complete or partial remission at the end of the first 3 months, this rate increased to 87% at the end of 12 months. Almost all of the patients were receiving steroid treatment. A study conducted in Italy revealed that patients receiving steroid treatment responded to the treatment at a rate of 80–90% [23]. Furthermore, one prospective study examined the effects of different types of steroid drugs, and it was found that their effects on long-term remission varied between 22–77% [24]. In a small-scale study, the response rate to IVIg treatment was found to be 75% [25]. Although the study results are similar to those obtained in our study, they differ from our study in that only first-line treatment results were considered in these studies. In our study, a mortality rate of 4% occurred over approximately 8 years. In a study conducted in the USA based on electronic records, the in-hospital mortality rate of ITP patients was found to be 3.8%, which is similar to the rate observed in our study [26]. In a cohort study, 5-, 10-, and 20-year mortality rates in adult patients diagnosed with ITP were found to be 22%, 34%, and 49%, respectively [27]. Causes of death among ITP patients are higher than in the general population due to cardiovascular disease, infection, bleeding, and hematological malignancy. It has been reported that immunosuppressant drugs used in the treatment of ITP also increase risks such as malignancy [28,29]. In our study, the shorter follow-up period may have caused the mortality rate to be lower. Mortality is also affected by ethnicity, age, and comorbidities. The platelet levels of the patients gradually increased in the 3rd and 12th months. In a study conducted in Hong Kong (*n* = 125), a permanent increase in platelet levels for 6 months was observed with steroid treatment alone [30]. IVIg has been indicated for combined use with steroids in the presence of severe bleeding and when the platelet count needs to be increased rapidly [31]. A systematic review showed that platelet levels peaked at a rate of 64–83% after IVIg treatment [32]. In our study, 81% of patients receiving IVIg treatment had complete or partial remission at the 3rd month and 88% at the 12th month. Although no significant difference was found between patients receiving and not receiving IVIg treatment, it has an important place in the treatment of ITP. IVIg is generally well-tolerated, with side effects occurring in approximately 5% of patients [33]. However, as IVIg is a plasma-derived product, it is a risk factor for the transmission of micro-organisms [34]. Furthermore, it should not be forgotten that both steroid and IVIg treatments may lead to serious side effects such as anaphylaxis or renal and pulmonary system damage. Thus, second-line drugs were also taken into consideration. 

Eltrombopag, a thrombopoietin receptor agonist (TPO-RA), was used in approximately one-quarter of the patients in our study. TPO-RAs are indicated in adult patients at risk of bleeding who have relapsed after splenectomy or who have contraindications to splenectomy [10]. Eltrombopag, which can be used alone or in combination with other second-line drugs, is a new-generation drug. A meta-analysis showed that eltrombopag caused an increase in platelet count with a relative risk of 3.4 [35]. In another study, it was associated with a response in platelet count in 60–80% of patients [36]. In our study, eltrombopag was used in approximately three-quarters of the patients. Partial or complete remission rates were over 65% in the 3rd and 12th months. In a retrospective study conducted in Spain, it was reported that complete remission was achieved with the use of eltrombopag in 77.3% of patients [37]. In a study examining the long-term efficacy and safety of Eltrombopag, it was shown that it is a well-tolerated, reliable drug which is effective in raising the platelet count [38]. Eltrombopag is the first TPO-r mimetic agent with a lower risk of side effects that can be used as an alternative to IVIg and steroids.

Complete remission occurred in 60–80% of patients using rituximab, which is a monoclonal antibody developed against CD20 antigen (anti-CD20) for the treatment of chronic and persistent ITP. It is an alternative treatment method in cases where splenectomy is contraindicated [17]. Studies have shown that the response to rituximab varies between 52–73%, and the complete remission rate varies between 20–54% [39]. A systematic review reported a 46.3% rate of complete remission in patients diagnosed with chronic ITP [40]. Although the rates were lower than those found in our study, the studies vary in terms of number of patients and study design. While half of the patients using danasin developed complete remission in the short-term, complete remission was not achieved in the long-term. A prospective study reported a 22% response rate to danazol treatment [41]. In a small-scale study (*n* = 9), an 11% response rate was obtained under danazol treatment despite side effects such as weight gain, joint pain, rash, and amenorrhea [42]. In another small-scale study (*n* = 10), it was reported that 10% responded to treatment; however, this response was temporary, and more than half of the patients developed side effects [43]. When the ITP treatment responses of immunosuppressive drugs are examined, it can be seen that the responses to cyclosporine and azathioprine are better in the short-term than cyclophosphamide and mycophenolate mofetil. In a retrospective study, the complete remission rate of azathioprine was shown to be 38% [44]. In two prospective studies, the complete remission rate with cyclophosphamide was found to be 50–65% [45,46]. In a retrospective study conducted in the pediatric age group, the complete remission rate of cyclosporine was reported to be 57% [47]. Studies on the remission rates of mycophenolate mofetil for ITP treatment have reported a complete remission rate of 24–45% [48,49,50].

In our study, we provide a pharmacological perspective on the approach to treatment and the treatment–response dilemma in ITP cases, which have recently increased among the population of patients living on the Mediterranean coastline. Emerging data regarding the use of second-line medical therapies in the treatment of patients with ITP requiring pharmacological intervention have led to a decrease in splenectomy rates. Thus, we can say that we have achieved the treatment/success rate observed in developed countries through early diagnosis and the use of new-generation immunosuppressive agents.

Similar to our study, the number of patients in the previous studies was low. More studies are needed to solidify the evidence regarding the effectiveness and safety of immunosuppressive drugs in the treatment of ITP. The small number of patients is one of the biggest limitations of this study. The fact that this study is retrospective and that first- and second-line treatments were applied in combination makes it difficult to make a comparison against drug therapy alone. However, as ITP is a rare disease and the number of clinical studies providing remission results with respect to treatment is low, our study contributes to the literature in this aspect.

## 5. Conclusions

Data of patients diagnosed with ITP receiving first- and second-line treatment in a training and research hospital were evaluated. In the study, the complete remission rates associated with drugs, especially those in the second-line drug category, were compared with those reported in similar studies in the literature, and the similarities and differences in the results were emphasized. It was noted that eltrombopag and rituximab, in particular, yield higher complete remission rates than immunosuppressive drugs. The use of new agents such as eltrombopag, which are easily tolerated and have a lower risk of side effects, is expected to increase, considering the side effects associated with immunosuppressive drugs, IVIg, and steroid therapy. However, as was the case in our study, the sample size used in studies conducted in the literature has been quite small. As such, randomized and blinded clinical trials with larger numbers of patients are needed.

## Figures and Tables

**Table 1:** Demographic and clinical characteristics of the study group (*n* = 100).

Demographic Features	
Age (mean ± standard deviation)	52.9 ± 19.6
Gender, *n* (%)	
Female	57
Male	43
Charlson Comorbidity Index, *n* (%)	
1–2 (mild)	67
3–4 (moderate)	10
≥5 (severe)	23
Information on ITP Diagnosis	
Time passed after diagnosis, *n* (%)	
≤4 year	47
5–7 years	32
≥8 years	21
Bleeding at diagnosis, *n* (%)	
Serious	3
Partial	95
None	2
Platelet transfusion in diagnosis, *n* (%)	4
Splenectomy, *n* (%)	41
Drugs	
Use of drugs in diagnosis, *n* (%)	
Antiplatelets	23
Anticoagulants	7
NSAIDs	11
First-line treatment, *n* (%)	
Methylprednisolone	99
IVIg	86
Second-line treatment, *n* (%)	
Eltrombopag	26
Rituximab	10
Danasin	4
Cyclosporine	3
Cyclophosphamide	1
Azathioprine	1
Mycophenolate Mofetil	1
Remissions	
Remission at the end of the 3rd month, *n* (%)	
Full	61
Partial	23
No response	16
Remission at the end of the 12th month, *n* (%)	
Full	73
Partial	17
No response	10

ITP: immune thrombocytopenia; NSAIDs: non-steroidal anti-inflammatory drugs; IVIg: intravenous immunoglobulin.

**Table 2:** ITP diagnosis (first admission) of the study group and laboratory findings in the 3rd and 12th months of treatment.

Hemogram	First Admission	3rd Month	12th Month
	Median (Q1–Q3)		
PLT (×10^3^/mL)	6 (3–17.75)	148.5 (54–264.3)	175 (69.3–286)
RBC (10^3^ mm^3^)	4.6 (4–5)	4.51 (4–5)	4.65 (4.3–5.1)
MCV (fL)	84.5 (80.25–88)	86 (83–89)	85.5 (81–89)
RDW (%)	14 (12.65–16)	14.75 (13–16)	15 (13–16.2)
MCH (pg/cell)	28 (26.25–29.97)	29 (27–30)	28 (27–30.1)
MCHC (gr/dL)	33 (32–34)	33 (32–34)	33 (32–34)
WBC (10^3^ mm^3^)	6.9 (5.5–8.85)	8.9 (6.75–12.11)	7.8 (6.16–10.73)
HGB (g/dL)	12.6 (11–14)	13 (11.42–14)	13 (12–14)
HCT (%)	38 (34–42)	39 (35–42.15)	39 (37–43)

Q1–Q3: interquartile range.

**Table 3:** Evaluation of remission rates according to first-line treatment.

*n*, %	3rd Month			12th Month		
	Full or Partial Remission (*n* = 84)	Non-Remission (*n* = 16)	*p* *	Full or Partial Remission (*n* = 90)	Non-Remission (*n* = 10)	*p* *
IVIg + (*n* = 86)	70 (81.4)	16 (18.6)	0.09	76 (88.4)	10 (11.6)	0.07
IVIg – (*n* = 14)	14 (100)	0 (0.0)		14 (100)	0 (0.0)	

*: Fisher’s exact test; IVIg: intravenous immunoglobulin.

**Table 4:** Evaluation of patients diagnosed with ITP and using drugs in second-line treatment.

No	Age	Gender	CCI	PLT (×10^3^/mL)	First-Line Treatment	Second-Line Treatment	3rd Month Remission	12th Month Remission
1	37	M	3	4	MP, IVIg	C, MM	-	-
2	29	M	0	5	MP, IVIg	E	Partial	Partial
3	35	M	0	2	MP, IVIg	R	Partial	Full
4	40	F	0	35	MP, IVIg	E, R	-	-
5	35	M	0	1	MP, IVIg	R	Partial	Full
6	79	M	7	39	MP, IVIg	E, D	-	-
7	54	M	1	1	MP, IVIg	E, R, C	Full	Full
8	76	M	7	2	MP, IVIg	E	Full	Full
9	31	F	0	8	MP, IVIg	E	Partial	Full
10	80	F	8	5	MP, IVIg	R	-	Full
11	85	F	6	8	MP, IVIg	R	Full	Full
12	88	M	5	1	MP, IVIg	E, D	Full	-
13	23	M	0	41	MP, IVIg	E	-	-
14	46	F	1	28	MP, IVIg	E	Full	Full
15	73	F	6	1	MP, IVIg	E, R	Full	Full
16	50	F	2	4	MP, IVIg	R	Full	Full
17	53	F	1	3	MP, IVIg	E	-	Full
18	40	M	2	3	MP, IVIg	E	-	Full
19	56	F	1	17	MP, IVIg	E	Full	Full
20	91	M	9	4	MP, IVIg	E, CP	-	Partial
21	93	F	9	22	IVIg	E	Full	Full
22	55	M	3	2	MP, IVIg	R	Full	Full
23	75	M	6	15	MP, IVIg	E, R	Full	Partial
24	85	F	8	3	MP, IVIg	E	-	Partial
25	66	M	2	5	MP, IVIg	E	Full	Full
26	61	F	6	2	MP, IVIg	E, D, A	Full	-
27	63	M	3	36	MP, IVIg	E	Full	Full
28	73	F	7	12	MP, IVIg	E, D	Partial	Partial
29	62	M	3	6	MP, IVIg	E	Partial	-
30	60	F	2	17	MP, IVIg	E	-	Partial

M: male; F: female; MP: methylprednisolone; IVIg: intravenous immunoglobulin; E: eltrombopag; R: rituximab; D: danasin; C: cyclosporine; CP: cyclophosphamide; A: azathioprine; MM: mycophenolate mofetil.

**Table 5:** Evaluation of megakaryocyte number/morphology in first-line treatment.

Morphological Properties	IVIG + (*n* = 45)	IVIG − (*n* = 9)	*p*
Megakaryocyte number, *n* (%)			
Increased	17 (89.5)	2 (10.5)	0.271 **
Normal	21 (87.5)	3 (12.5)	
Decreased	5 (62.5)	3 (37.5)	
Not evaluated	2 (66.7)	1 (33.3)	
Hypolobulation, *n* (%)	12 (92.3)	1 (7.7)	0.428 **
Hyperlobulation, *n* (%)	8 (88.9)	1 (11.1)	1.000 **

**: Fisher’s exact test.

**Table 6:** Evaluation of megakaryocyte number/morphology in second-line treatment.

Morphological Properties	Secondary Drug Use − (*n* = 33)	Secondary Drug Use + (*n* = 21)	*p*
Megakaryocyte number, *n* (%)			
Increased	7 (36.8)	12 (63.2)	0.487 **
Normal	11 (45.8)	13 (54.2)	
Decreased	3 (37.5)	5 (62.5)	
Not evaluated	0 (0.0)	3 (100.0)	
Hypolobulation, *n* (%)	8 (61.5)	5 (38.5)	0.971 **
Hyperlobulation, *n* (%)	5 (55.6)	4 (44.4)	0.723 **

**: Fisher’s exact test.

## Data Availability

The data that support the findings of this study are available from the corresponding author upon reasonable request.

## References

[1] Liu X., Hou Y., Hou M. (2023). How We Treat Primary Immune Thrombocytopenia in Adults. J. Hematol. Oncol..

[2] González-López T.J., Provan D., Bárez A., Bernardo-Gutiérrez A., Bernat S., Martínez-Carballeira D., Jarque-Ramos I., Soto I., Jiménez-Bárcenas R., Fernández-Fuertes F. (2023). Primary and Secondary Immune Thrombocytopenia (ITP): Time for a Rethink. Blood Rev..

[3] Boulware R., Refaai M.A. (2020). Why Do Patients with Immune Thrombocytopenia (ITP) Experience Lower Bleeding Events despite Thrombocytopenia?. Thromb. Res..

[4] Moulis G., Comont T., Adoue D. (2021). New Insights into the Epidemiology of Immune Thrombocytopenia in Adult Patients: Impact for Clinical Practice. Rev. Méd. Interne.

[5] Lee J.Y., Lee J.-H., Lee H., Kang B., Kim J.-W., Kim S.H., Lee J.-O., Kim J.W., Kim Y.J., Lee K.-W. (2017). Epidemiology and Management of Primary Immune Thrombocytopenia: A Nationwide Population-Based Study in Korea. Thromb. Res..

[6] Crickx E., Mahévas M., Michel M., Godeau B. (2023). Older Adults and Immune Thrombocytopenia: Considerations for the Clinician. Clin. Interv. Aging.

[7] Grimaldi-Bensouda L., Nordon C., Michel M., Viallard J.-F., Adoue D., Magy-Bertrand N., Durand J.-M., Quittet P., Fain O., Bonnotte B. (2016). Immune Thrombocytopenia in Adults: A Prospective Cohort Study of Clinical Features and Predictors of Outcome. Haematologica.

[8] Tripathi A.K., Mishra S., Kumar A., Yadav D., Shukla A., Yadav Y. (2014). Megakaryocyte Morphology and Its Impact in Predicting Response to Steroid in Immune Thrombocytopenia. Platelets.

[9] Godeau B. (2014). Immune Thrombocytopenic Purpura: Major Progress in Knowledge of the Pathophysiology and the Therapeutic Strategy, but Still a Lot of Issues. Presse Med..

[10] Rodeghiero F., Stasi R., Gernsheimer T., Michel M., Provan D., Arnold D.M., Bussel J.B., Cines D.B., Chong B.H., Cooper N. (2009). Standardization of Terminology, Definitions and Outcome Criteria in Immune Thrombocytopenic Purpura of Adults and Children: Report from an International Working Group. Blood.

[11] Vrbensky J.R., Nazy I., Toltl L.J., Ross C., Ivetic N., Smith J.W., Kelton J.G., Arnold D.M. (2018). Megakaryocyte Apoptosis in Immune Thrombocytopenia. Platelets.

[12] Neunert C., Terrell D.R., Arnold D.M., Buchanan G., Cines D.B., Cooper N., Cuker A., Despotovic J.M., George J.N., Grace R.F. (2019). American Society of Hematology 2019 Guidelines for Immune Thrombocytopenia. Blood Adv..

[13] Piel-Julian M.-L., Mahévas M., Germain J., Languille L., Comont T., Lapeyre-Mestre M., Payrastre B., Beyne-Rauzy O., Michel M., Godeau B. (2018). Risk Factors for Bleeding, Including Platelet Count Threshold, in Newly Diagnosed Immune Thrombocytopenia Adults. J. Thromb. Haemost..

[14] Adelborg K., Kristensen N.R., Nørgaard M., Bahmanyar S., Ghanima W., Kilpatrick K., Frederiksen H., Ekstrand C., Sørensen H.T., Fynbo Christiansen C. (2019). Cardiovascular and Bleeding Outcomes in a Population-based Cohort of Patients with Chronic Immune Thrombocytopenia. J. Thromb. Haemost..

[15] Hamzah R., Yusof N., Tumian N.R., Abdul Aziz S., Mohammad Basri N.S., Leong T.S., Ho K.W., Selvaratnam V., Tan S.M., Muhamad Jamil S.A. (2022). Clinical Epidemiology, Treatment Outcome and Mortality Rate of Newly Diagnosed Immune Thrombocytopenia in Adult Multicentre Study in Malaysia. J. Blood Med..

[16] Arnold D.M. (2015). Bleeding Complications in Immune Thrombocytopenia. Hematology.

[17] Neunert C., Lim W., Crowther M., Cohen A., Solberg L., Crowther M.A. (2011). The American Society of Hematology 2011 Evidence-Based Practice Guideline for Immune Thrombocytopenia. Blood.

[18] Provan D., Arnold D.M., Bussel J.B., Chong B.H., Cooper N., Gernsheimer T., Ghanima W., Godeau B., González-López T.J., Grainger J. (2019). Updated International Consensus Report on the Investigation and Management of Primary Immune Thrombocytopenia. Blood Adv..

[19] Gómez-Almaguer D. (2018). Eltrombopag-Based Combination Treatment for Immune Thrombocytopenia. Ther. Adv. Hematol..

[20] Zhu J., Chen R., Zhao S., Zhu L., Li X., Xie M., Ye X. (2020). A Megakaryocyte Morphological Classification-Based Predictive Model for Steroid Sensitivity in Primary Immune Thrombocytopenia. Platelets.

[21] Tripathi A.K., Shukla A., Mishra S., Yadav Y.S., Yadav D.K. (2014). Eltrombopag Therapy in Newly Diagnosed Steroid Non-Responsive ITP Patients. Int. J. Hematol..

[22] Charlson M.E., Pompei P., Ales K.L., MacKenzie C.R. (1987). A New Method of Classifying Prognostic Comorbidity in Longitudinal Studies: Development and Validation. J. Chronic Dis..

[23] Mazzucconi M.G., Fazi P., Bernasconi S., De Rossi G., Leone G., Gugliotta L., Vianelli N., Avvisati G., Rodeghiero F., Amendola A. (2007). Therapy with High-Dose Dexamethasone (HD-DXM) in Previously Untreated Patients Affected by Idiopathic Thrombocytopenic Purpura: A GIMEMA Experience. Blood.

[24] Matschke J., Müller-Beissenhirtz H., Novotny J., Vester I., Hertenstein B., Eisele L., Lax H., Ose C., Dührsen U. (2016). A Randomized Trial of Daily Prednisone versus Pulsed Dexamethasone in Treatment-Naïve Adult Patients with Immune Thrombocytopenia: EIS 2002 Study. Acta Haematol..

[25] Mayer B., Depré F., Ringel F., Salama A. (2017). New Aspects on the Efficacy of High-dose Intravenous Immunoglobulins in Patients with Autoimmune Thrombocytopenia. Vox Sang..

[26] Danese M.D., Lindquist K., Gleeson M., Deuson R., Mikhael J. (2009). Cost and Mortality Associated with Hospitalizations in Patients with Immune Thrombocytopenic Purpura. Am. J. Hematol..

[27] Frederiksen H., Maegbaek M.L., Nørgaard M. (2014). Twenty-year Mortality of Adult Patients with Primary Immune Thrombocytopenia: A Danish Population-based Cohort Study. Br. J. Haematol..

[28] Marcén R. (2009). Immunosuppressive Drugs in Kidney Transplantation. Drugs.

[29] Sorensen H.T., Mellemkjaer L., Nielsen G.L., Baron J.A., Olsen J.H., Karagas M.R. (2004). Skin Cancers and Non-Hodgkin Lymphoma among Users of Systemic Glucocorticoids: A Population-Based Cohort Study. JNCI J. Natl. Cancer Inst..

[30] Cheng Y., Wong R.S.M., Soo Y.O.Y., Chui C.H., Lau F.Y., Chan N.P.H., Wong W.S., Cheng G. (2003). Initial Treatment of Immune Thrombocytopenic Purpura with High-Dose Dexamethasone. N. Engl. J. Med..

[31] Cooper N., Ghanima W. (2019). Immune Thrombocytopenia. N. Engl. J. Med..

[32] Bussel J.B., Pham L.C. (1987). Intravenous Treatment with Gammaglobulin in Adults with Immune Thrombocytopenic Purpura: Review of the Literature. Vox Sang..

[33] Abadi U., Yarchovsky-Dolberg O., Ellis M.H. (2015). Immune Thrombocytopenia. Clin. Appl. Thromb..

[34] Arnold D.M. (2013). Positioning New Treatments in the Management of Immune Thrombocytopenia. Pediatr. Blood Cancer.

[35] Elgebaly A.S., Ashal G.E., Elfil M., Menshawy A. (2017). Tolerability and Efficacy of Eltrombopag in Chronic Immune Thrombocytopenia: Meta-Analysis of Randomized Controlled Trials. Clin. Appl. Thromb..

[36] Cheng G., Saleh M.N., Marcher C., Vasey S., Mayer B., Aivado M., Arning M., Stone N.L., Bussel J.B. (2011). Eltrombopag for Management of Chronic Immune Thrombocytopenia (RAISE): A 6-Month, Randomised, Phase 3 Study. Lancet.

[37] González-López T.J., Pascual C., Álvarez-Román M.T., Fernández-Fuertes F., Sánchez-González B., Caparrós I., Jarque I., Mingot-Castellano M.E., Hernández-Rivas J.A., Martín-Salces M. (2015). Successful Discontinuation of Eltrombopag after Complete Remission in Patients with Primary Immune Thrombocytopenia. Am. J. Hematol..

[38] Wong R.S.M., Saleh M.N., Khelif A. (2017). Safety and Efficacy of Long-Term Treatment of Chronic/Persistent ITP with Eltrombopag: Final Results of the EXTEND Study. Blood.

[39] Lucchini E., Zaja F., Bussel J. (2019). Rituximab in the Treatment of Immune Thrombocytopenia: What Is the Role of This Agent in 2019?. Haematologica.

[40] Arnold D.M., Dentali F., Crowther M.A., Meyer R.M., Cook R.J., Sigouin C., Fraser G.A., Lim W., Kelton J.G. (2007). Systematic Review: Efficacy and Safety of Rituximab for Adults with Idiopathic Thrombocytopenic Purpura. Ann. Intern. Med..

[41] Bourgeois E., Caulier M.T., Delarozee C., Brouillard M., Bauters F., Fenaux P. (2003). Long-term Follow-up of Chronic Autoimmune Thrombocytopenic Purpura Refractory to Splenectomy: A Prospective Analysis. Br. J. Haematol..

[42] Almagro D. (1985). Danazol in Idiopathic Thrombocytopenic Purpura. Acta Haematol..

[43] Mazzucconi M.G., Francesconi M., Falcione E., Ferrari A., Gandolfo G.M., Ghirardini A., Tirindelli M.C. (1987). Danazol Therapy in Refractory Chronic Immune Thrombocytopenic Purpura. Acta Haematol..

[44] Chang H., Tang T., Hung Y., Li P., Kuo M., Wu J., Wang P. (2018). Immune Thrombocytopenia: Effectiveness of Frontline Steroids and Comparison of Azathioprine, Splenectomy, and Rituximab as Second-line Treatment. Eur. J. Haematol..

[45] Reiner A., Gernsheimer T., Slichter S. (1995). Pulse Cyclophosphamide Therapy for Refractory Autoimmune Thrombocytopenic Purpura [See Comments]. Blood.

[46] Verlin M., Laros R.K., Penner J.A. (1976). Treatment of Refractory Thrombocytopenic Purpura with Cyclophosphamide. Am. J. Hematol..

[47] Liu A.P.Y., Cheuk D.K.L., Lee A.H.Y., Lee P.P.W., Chiang A.K.S., Ha S.Y., Tsoi W.C., Chan G.C.F. (2016). Cyclosporin A for Persistent or Chronic Immune Thrombocytopenia in Children. Ann. Hematol..

[48] Zhang W., Ji L., Cao X., Chen Y., He A., Liu J., Zhao W., Zou S. (2005). Mycophenolate Mofetil as a Treatment for Refractory Idiopathic Thrombocytopenic Purpura. Acta Pharmacol. Sin..

[49] Hou M., Peng J., Shi Y., Zhang C., Qin P., Zhao C., Ji X., Wang X., Zhang M. (2003). Mycophenolate Mofetil (MMF) for the Treatment of Steroid-resistant Idiopathic Thrombocytopenic Purpura. Eur. J. Haematol..

[50] Taylor A., Neave L., Solanki S., Westwood J.P., Terrinonive I., McGuckin S., Kothari J., Cooper N., Stasi R., Scully M. (2015). Mycophenolate Mofetil Therapy for Severe Immune Thrombocytopenia. Br. J. Haematol..

